# MICROLEVEL COMMUNICATION BETWEEN FAMILY CAREGIVERS AND PERSONS LIVING WITH DEMENTIA

**DOI:** 10.1093/geroni/igac059.1587

**Published:** 2022-12-20

**Authors:** Sohyun Kim, Wen Liu

**Affiliations:** University of Massachusetts, Amherst, Massachusetts, United States; University of Iowa, Iowa City, Iowa, United States

## Abstract

Little is known about the relationship between family caregivers and persons living with dementia communication at micro-level (smallest verbal and nonverbal behavior unit). Micro-level communication was assessed second-by-second using 75 in-home video recordings from 19 caregiver-care recipient dyads. Each care recipient (N = 38) and caregiver (N = 42) behavior unit was paired and compared using Spearman’s partial correlation. Two-hundred twenty care recipient-caregiver behavior unit pairs were correlated (n = 148 verbal, n = 72 nonverbal pairs, rs range = .456 - .990, all p < .05). Ninety-one caregiver facilitative-care recipient engaging behavior unit pairs (e.g., comfort/empathetic touch, using humor) were positively correlated (rs range = .460 - .990, all p < .05). Eighteen caregiver disabling-care recipient challenging behavior unit pairs (e.g., withdrawing) were positively correlated (rs range = .456 - .697, all p < .05). Individualized caregiver education for matching and adjusting communication is needed to facilitate meaningful interaction.

